# A Multiyear Survey and Identification of Pepper- and Tomato-Infecting Viruses in Yunnan Province, China

**DOI:** 10.3389/fmicb.2021.623875

**Published:** 2021-02-26

**Authors:** Yueyue Li, Guanlin Tan, Long Xiao, Wenpeng Zhou, Pingxiu Lan, Xiaojiao Chen, Yong Liu, Ruhui Li, Fan Li

**Affiliations:** ^1^State Key Laboratory for Conservation and Utilization of Bio-Resources in Yunnan, Yunnan Agricultural University, Kunming, China; ^2^College of Life Science, Luoyang Normal University, Luoyang, China; ^3^Modern Education Technology Center, Yunnan Agricultural University, Kunming, China; ^4^Hunan Plant Protection Institute, Hunan Academy of Agricultural Sciences, Changsha, China; ^5^USDA-ARS, National Germplasm Resources Laboratory, Beltsville, MD, United States

**Keywords:** pepper, tomato, virus disease, virus identification, mixed infection

## Abstract

During pepper and tomato production seasons in 2013–2017, large-scale virus disease surveys were conducted in different regions of Yunnan Province, China. A total of 1,267 pepper and tomato samples with various virus-like symptoms were collected and analyzed for virus infections through dot enzyme-linked immunosorbent assay (dot-ELISA), polymerase chain reaction (PCR), and reverse-transcription (RT)-PCR. The detection results showed that 19 different viruses were present in about 50.9% of the assayed samples, and among these viruses, seven viruses were found in both pepper and tomato samples. Mixed infections with two to three of the 15 identified mixed infection types were found in the pepper samples and 10 identified mixed infection types were found in the tomato samples. Among the infected samples, *Tomato spotted wilt orthotospovirus* (TSWV) was the most common virus, with a detection rate of about 20.0% followed by *Pepper vein yellows virus* (PeVYV, 13.0%). This survey revealed for the first time that pepper is a natural host of *Tobacco vein distorting virus* (TVDV) worldwide and tomato is a natural host of *Potato leafroll virus* (PLRV) in China. PeVYV, *Tobacco mild green mosaic virus* (TMGMV) and *Wild tomato mosaic virus* (WTMV) were first time found in pepper and *Tomato mottle mosaic virus* (ToMMV) and *Chilli veinal mottle virus* (ChiVMV) were first time found in tomato in Yunnan Province. Finally, the virus incidences were higher in Kunming, Yuxi, Chuxiong, and Honghe region than other regions.

## Introduction

Pepper (*Capsicum annuum*) and tomato (*Solanum lycopersicum*) are two economically important solanaceous vegetable crops in China and many other countries. With the rapid increase of production areas and growth of new cultivars in the recent years, outbreaks of virus disease have become common in solanaceous crops, leading to serious yield losses. Approximately 76 and 136 viruses have been reported for pepper and tomato plants throughout the world, respectively (Dong et al., 2008; Hanssen et al., 2010; Kenyon et al., 2014; Li et al., 2014; Feng et al., 2017; Mauricio-Castillo et al., 2017; Olaya et al., 2017; Wang et al., 2017a; Liu et al., 2019). To date, 28 RNA and three DNA viruses have been reported for pepper crops, and 22 RNA and 12 DNA viruses have been reported for tomato crops in China. Many studies have suggested that *Cucumber mosaic virus* (CMV) and *Tobacco mosaic virus* (TMV) were the most predominant viruses in both pepper and tomato fields in China (Dong et al., 2008; Tao and Zhou, 2008; Zhang et al., 2009, 2017a; Qing et al., 2010; Wang and Huang, 2010; Zhang, 2010; Ge et al., 2012; Ruan, 2012; Ding et al., 2013; Li et al., 2014; Wang et al., 2014, 2017b; Zhao et al., 2014; Guo et al., 2015; Padmanabhan et al., 2015; Yuan et al., 2015; Feng et al., 2017; Liu et al., 2019).

Yunnan Province is located at the southwest border of China, neighboring Laos, Myanmar, and Vietnam. The low-latitude and high-elevation geographic features create a multiclimate weather, suitable for many vegetable productions, including many solanaceous vegetable crops. The total vegetable production acreage in Yunnan Province in 2017 had reached about 1.1 million ha and produced approximately 20.78 million tons of vegetables products, valued about 7.15 billion United States dollars. Pepper and tomato are the two major solanaceous vegetable crops in Yunnan Province. Due to virus diseases, some pepper and tomato fields in this province had total yield losses in the recent years, resulting in a quick increase of pesticide applications, insect tolerance to pesticides, and environment pollutions (Chen and Liu, 2013; Bu et al., 2014).

Identification of viruses and their epidemiology in pepper and tomato fields are critical for the development of effective managements for the viruses. Although field surveys of virus diseases in pepper and tomato fields had been reported for other provinces of China (Wen et al., 2009; Wang et al., 2014, 2017b; Guo et al., 2015; Liu, 2015; Wang L. S. et al., 2015; Gao et al., 2016; Zhang et al., 2017b), similar survey has not been done in Yunnan Province. Through this study, we have found that virus infections in these two crops are more common in Yunnan Province than that in other Chinese provinces.

## Materials and Methods

### Field Survey and Sample Collection

Leaves and fruits showing virus-like symptoms were first recorded using a Sony camera (DSC-RX100M5, Sony), harvested, and then stored separately in plastic sampling bags till use. This survey focused on pepper and tomato crops in 38 counties belonging to 12 different cities or autonomous regions from April to October in 2013, 2014, 2015, and 2016 and April in 2017. More tissue samples were collected from the surveyed fields with higher disease incidences and severer disease symptoms, while fewer samples were collected from the fields with lower disease incidences and milder disease symptoms (Table 1). The collected samples were analyzed for virus infections immediately or stored at −80°C or −20°C until use.

**TABLE 1:** Numbers of pepper and tomato samples collected from different regions of Yunnan Province.

Sampling sites		Number of samples	
City/Autonomous region	County	Pepper	Tomato
Chuxiong	Yuanmou, Shuangbai, Yaoan, Nanhua, Lufeng, Chuxiong	116	190
Yuxi	Hongta, Jiangchuan, Tonghai, Chengjiang, Huaning, Xinping	135	22
Honghe	Shiping, Jianshui, Kaiyuan, Mengzi	212	60
Kunming	Panlong, Dongchuan, Yiliang, Xundian, Songming, Jinning	140	97
Wenshan	Wenshan, Guangnan, Yanshan, Qiubei	89	25
Dehong	Mangshi	11	21
Dali	Dali	28	0
Baoshan	Shidian, Longling	51	6
Lijiang	Gucheng	10	22
Zhaotong	Zhaoyang	13	0
Qujing	Shizong, Luliang, Huize, Xuanwei	13	3
Puer	Ninger, Jiangcheng	3	0
Total number		821	446

### Serological Tests

Serological tests were done using the dot enzyme-linked immunosorbent assay (dot-ELISA) as described (Xie et al., 2013) with slight modifications. Briefly, each sample (0.1 g) was placed in a precooled 2-ml tube with five 3 mm and one 6 mm stainless-steel beads. After addition of 1 ml 0.01 M phosphate-buffered saline (PBS, 0.13 M NaCl, 0.003 M KCl, 0.008 M Na_2_HPO_4_, 0.001 M KH_2_PO_4_, pH 7.4), the tube was placed at −80°C for 5 min, and then ground using a high-throughput tissue homogenizer (Scientz-48, Ningbo Scientz) set at 60 Hz for 2 × 90 s (twice). The tube was centrifuged at 8,000 × *g* and 4°C for 5 min, and the supernatant in each tube was blotted onto a nitrocellulose membrane (2 μl per dot, three dots per sample) followed by air-drying. The membrane was incubated for 30 min in 10 ml PBST buffer (0.01 M PBS containing 0.05% Tween-20, pH 7.4) supplemented with 5% skim milk powder and then probed with a diluted monoclonal antibody for 1 h at 37°C. Monoclonal antibodies specific for *Broad bean wilt virus 2* (BBWV2), *Cucumber green mottle mosaic virus* (CGMMV), CMV, TMV, *Tomato spotted wilt orthotospovirus* (TSWV), or *Turnip mosaic virus* (TuMV) were kindly provided by Prof. Xueping Zhou, Zhejiang University, Hangzhou, China. After four washes in the PBST solution, the membrane was incubated again in 10 ml 10,000 times diluted alkaline phosphatase (AP)-conjugated goat anti-mouse IgG (Sigma-Aldrich, St. Louis, MO, USA) in PBST for 1 h at 37°C. After five washes in PBST, the membrane was incubated in 5 ml AP substrate solution [0.1 M Tris, pH 9.5, 0.1 M NaCl and 0.025 M MgCl_2_, supplemented with nitro-blue tetrazolium chloride/5-bromo-4-chloro-3-indolyl phosphate (Sangon Biotech., Shanghai, China)] as instructed for color development at room temperature for 10–25 min. The reaction was terminated after the positive control dots developed a purple color, whereas the negative control dots remained a light green color. A sample was considered infected if all three dots developed the purple color. Positive control samples representing BBWV2, CMV, TMV, TSWV, or TuMV were maintained in our laboratory, and the CGMMV-infected control sample was kindly provided by Dr. Zhaobang Cheng, Jiangsu Academy of Agricultural Science, Nanjing, China.

### Total Nucleic Acid Extraction, Polymerase Chain Reaction and Reverse Transcription-PCR

Total nucleic acid was extracted from tissue samples using a modified CTAB method (Li et al., 2008a). Degenerated and specific primers for polymerase chain reaction (PCR) or reverse transcription (RT)-PCR were as reported (Ha et al., 2006, 2008; Hirota et al., 2010; Zhou et al., 2011; Zhang et al., 2017b; Li et al.,2018a,b,c, 2020; Liu et al., 2019) or made in this study (Supplementary Table 1). First-strand cDNA was synthesized using the Reverse Transcriptase M-MLV (RNase H^–^) Kit (TaKaRa Biotech, Dalian, China). Each RT reaction contained about 200 ng of total RNA (2 μl), 1 μl of Random Oligo-dT (N6) primer (TaKaRa Biotech., Dalian, China) or a specific reverse primer (10 μM), and 3 μl nuclease-free ddH_2_O. The mixture was incubated at 70°C for 10 min and then chilled immediately on ice for 3 min. After addition of 2 μl of 5 × M-MLV buffer, 0.5 μl of dNTP mixture (10 mM each), 0.5 μl of RTase M-MLV (RNase H^–^), and 1 μl of nuclease-free H_2_O, the reaction mixture was incubated at 42°C for 1 h followed by 70°C for 15 min. Each PCR reaction was 10 μl (5 μl Green Taq Mix (Vazyme Biotech. Co., Ltd., China), 0.2 μl each primer (10 μM each), 1 μl cDNA (RT-PCR) or total nucleic acid (PCR), and 3.6 μl nuclease-free H_2_O), and the reaction condition was initial denaturation at 94°C for 5 min, 35 cycles (for reactions using specific primers) or 40 cycles (for reactions using degenerated primers) of 94°C for 30 s, 40–55°C for 30 s, and 72°C for 1 min, followed by the final extension at 72°C for 7 min. The PCR or RT-PCR products were visualized in 1% agarose gels through electrophoresis in 0.5 × TBE buffer. The resulting agarose gels were stained with 5 μl Gold View as instructed (Solarbio Science & Technology Co., Ltd., Beijing, China) and examined under an UV illumination GenoSens1850 system.

### Cloning and DNA Sequencing

PCR products were recovered using a Universal DNA Purification Kit (TIANGEN Biotech., Beijing, China) and then cloned into the pMD19-T Vector (TaKaRa Biotech., Dalian, China). At least three clones representing a single PCR product were sequenced by the Beijing Genomics Institute (BGI, Shenzhen, China). The resulting sequences were used to BLAST search the sequence database at the National Center for Biotechnology Information^1^. The results were then confirmed by both dot-ELISA and RT-PCR for RNA viruses or PCR for DNA viruses.

## Results

### Field Survey Result

**FIGURE 1:**
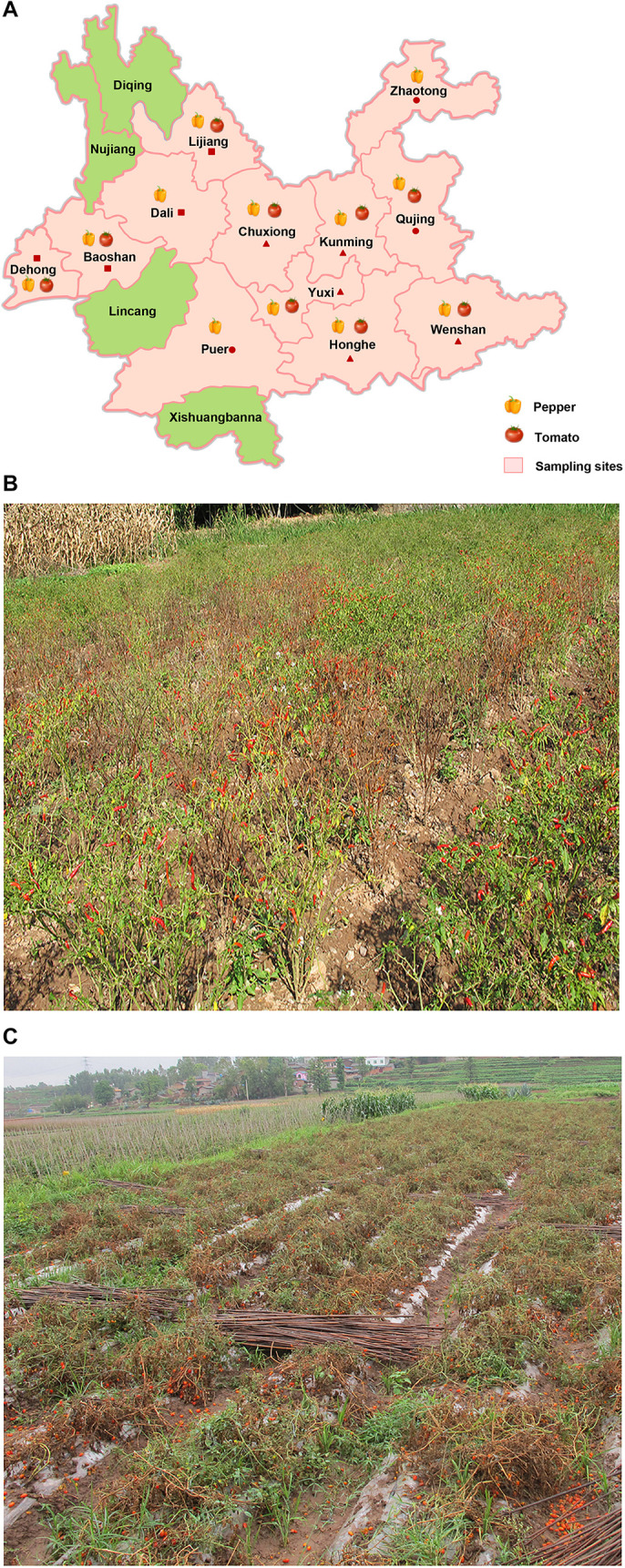
Virus detection using field-collected pepper and tomato samples in Yunnan Province. **(A)** Distribution of sampling sites in this study is shown. The sampling sites are shown in pink, and pepper and tomato images indicate the samples collected in the indicated region. The sampling regions with a triangle symbol are the regions with more samples collected and with more fields having higher virus disease incidences. The sampling regions with a square symbol are the regions with less than 100 samples collected and with fewer fields having common virus infections. The sampling regions with a circular symbol are the regions with only a few samples collected and the infected plants were only sporadic. Representative pepper **(B)** and tomato **(C)** fields with near 100% TSWV infection. These photos were taken in Honghe Autonomous region in 2013 **(B)** and Chuxiong Autonomous region in 2014 **(C)**.

Pepper and tomato fields in 38 counties, belonging to 12 different cities or autonomous regions (refer to as regions thereafter) were surveyed for virus diseases in 2013, 2014, 2015, 2016, and 2017 (Table 1 and Figure 1A). A total number of 821 pepper samples and 446 tomato samples were collected during the surveys. The most common symptoms on the infected pepper and tomato plants were mottling or mosaic, necrosis, yellowing, and distortion in leaves. The survey results showed that most of the surveyed pepper and tomato fields in Chuxiong, Yuxi, Honghe, and Kunming regions had a near 100% virus disease incidence, resulting in complete production failures (Figures 1B,C). Most surveyed fields in Wenshan, Dehong, Dali, and Baoshan regions had 10–25% virus disease incidences, while diseased plants in most surveyed fields in the Lijiang, Zhaotong, Qujing, and Puer regions were sporadic.

### Virus Diversity

**FIGURE 2:**
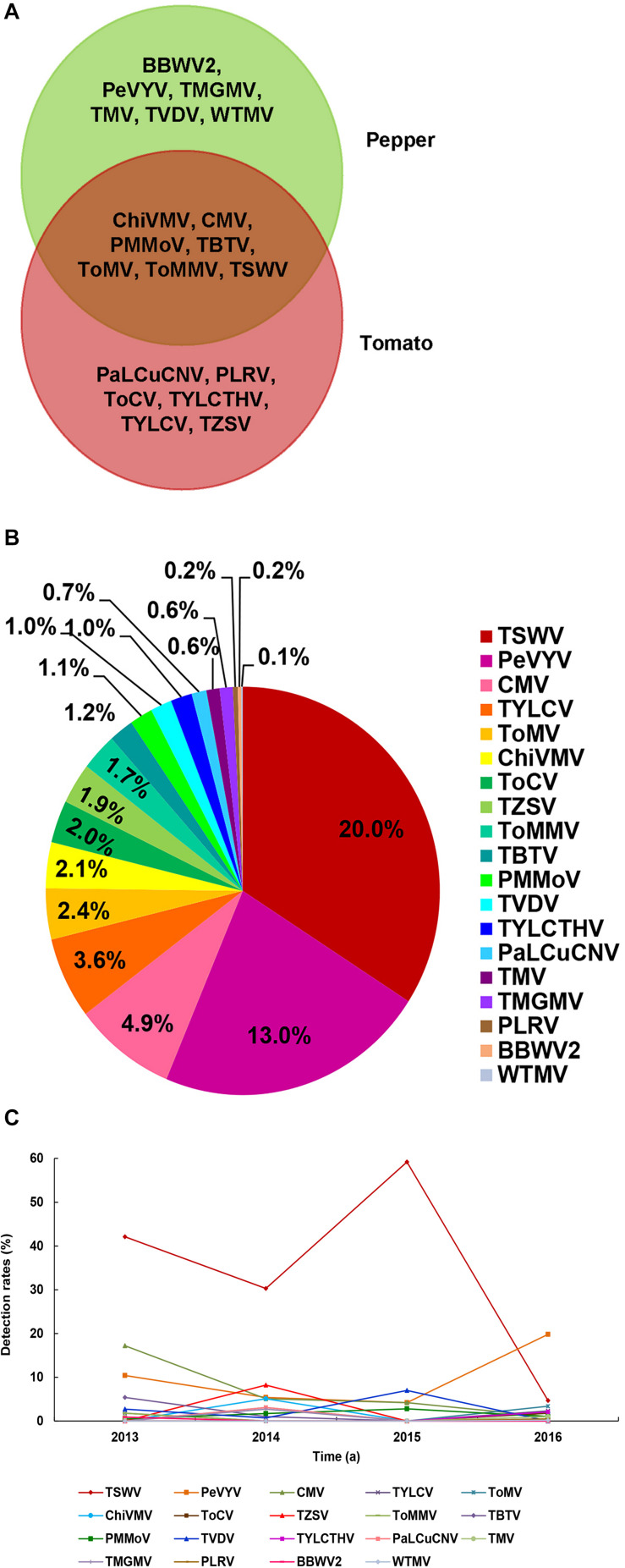
Virus population structures in pepper and tomato fields in Yunnan Province. **(A)** Viruses identified in the pepper and tomato samples. The viruses identified in the pepper samples is shown in the green area, the viruses identified in tomato samples is shown in the red area, and the viruses identified in both pepper and tomato samples is shown in the overlapped area. **(B)** Detection rates of viruses in pepper and tomato samples. Abbreviation of each detected virus is represented with a defined color, and the detection rate of each virus is marked in the corresponding icon. **(C)** Occurrence of different viruses from 2013 to 2016. Abbreviation of each detected virus is represented by a defined color legend line.

A total number of 19 different viruses were detected in 645 of the total 1,267 samples (50.9%) (Table 2). Among these viruses, *Chilli veinal mottle virus* (ChiVMV), CMV, *Pepper mild mottle virus* (PMMoV), *Tobacco bushy top virus* (TBTV), *Tomato mosaic virus* (ToMV), *Tomato mottle mosaic virus* (ToMMV), and TSWV were detected in both pepper and tomato samples (Figure 2A). The virus detection result also showed that TSWV was the most common virus (253 out of 1,267 assayed samples) in Yunnan Province, followed by *Pepper vein yellows virus* (PeVYV) and CMV (Figure 2B and Table 2). Further assays showed that virus population structures in the pepper and tomato fields in these regions varied each year (Figure 2C). For instance, unlike other viruses, TSWV, PeVYV, CMV, and PMMoV were detected in each year in the surveyed pepper and tomato fields, and the incidence of TSWV in 2013, 2014, 2015, and 2016 was at 42.1, 30.3, 59.2, and 4.7%, respectively (Table 2 and Figure 2C).

**TABLE 2:** Virus detection rates on pepper and tomato in different years in Yunnan.

Virus	Virus abbreviation	Detection rate in different years (%)				
		2013	2014	2015	2016	2017
*Tomato spotted wilt orthotospovirus*	TSWV	42.1	30.3	59.2	4.7	0.0
*Pepper vein yellows virus*	PeVYV	10.4	5.4	4.2	19.8	0.0
*Cucumber mosaic virus*	CMV	17.2	5.1	4.2	1.0	0.0
*Tomato yellow leaf curl virus*	TYLCV	1.8	0.0	0.0	2.3	47.5
*Tomato mosaic virus*	ToMV	0.0	3.1	0.0	3.4	0.0
*Chilli veinal mottle virus*	ChiVMV	0.0	5.1	0.0	1.9	0.0
*Tomato chlorosis virus*	ToCV	0.0	0.0	0.0	1.8	23.7
*Tomato zonate spot orthotospovirus*	TZSV	0.0	8.2	0.0	0.0	0.0
*Tomato mottle mosaic virus*	ToMMV	1.8	0.0	0.0	1.0	20.3
*Tobacco bushy top virus*	TBTV	5.4	1.0	0.0	0.0	0.0
*Pepper mild mottle virus*	PMMoV	0.5	1.7	2.8	1.0	0.0
*Tobacco vein distorting virus*	TVDV	2.7	0.7	7.0	0.0	0.0
*Tomato yellow leaf curl Thailand virus*	TYLCTHV	0.0	0.0	0.0	2.1	0.0
*Papaya leaf curl China virus*	PaLCuCNV	0.0	3.1	0.0	0.0	0.0
*Tobacco mosaic virus*	TMV	0.9	0.0	0.0	1.0	0.0
*Tobacco mild green mosaic virus*	TMGMV	0.0	2.7	0.0	0.0	0.0
*Potato leaf roll virus*	PLRV	0.0	0.0	0.0	0.5	0.0
*Broad bean wilt virus* 2	BBWV2	0.9	0.0	0.0	0.0	0.0
*Wild tomato mosaic virus*	WTMV	0.0	0.0	0.0	0.2	0.0

### Geographical Distributions of Viruses

**FIGURE 3:**
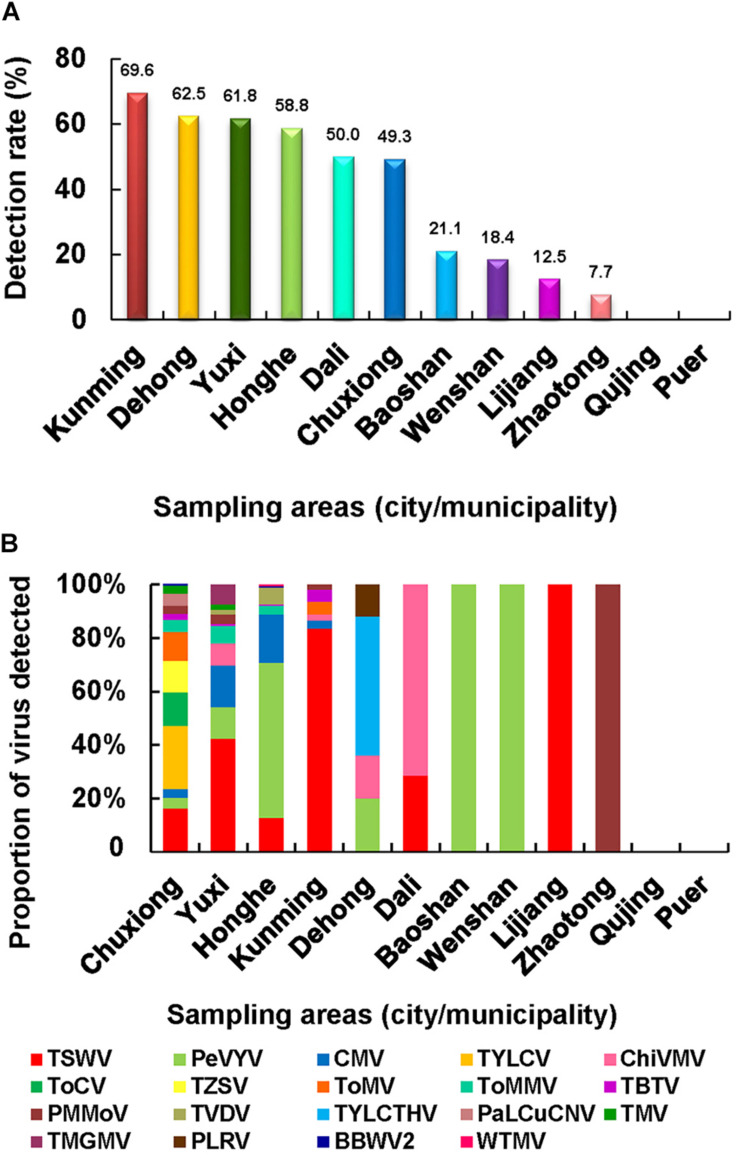
Virus population structures in different surveyed regions. **(A)** Virus detection rates in the 12 surveyed regions. **(B)** Virus population structures in different regions surveyed in this study.

To determine the geographical distributions of these viruses in Yunnan Province, we analyzed the field survey and virus detection data. The result showed that the virus detection rates in Kunming, Yuxi, Honghe, and Chuxiong regions were higher, ranging from 49.3 to 69.6% (Figure 3A), and also had much higher virus incidences in fields. Dehong and Dali regions also had high virus detection rates (62.5 and 50.0%, respectively), but the virus incidences in fields were not high; their high virus detection rates were due possibly to the less number of samples. For virus population structures, a total of 13 viruses were detected in the pepper and tomato samples collected from Chuxiong region, followed by 10 viruses in the samples from Yuxi region, eight viruses in the samples from Honghe region, six viruses in the samples from Kunming region, four viruses in the samples from Dehong region, and two viruses in the samples from Dali region (Figure 3B). Only one virus was detected in the samples from Baoshan, Wenshan, Lijiang, and Zhaotong regions. No virus was found in the samples from Qujing and Puer regions (Figure 3A), where lower virus incidence and small sample sizes may be responsible for the failure of virus detection.

### Distributions of Pepper-Infecting Viruses

**FIGURE 4:**
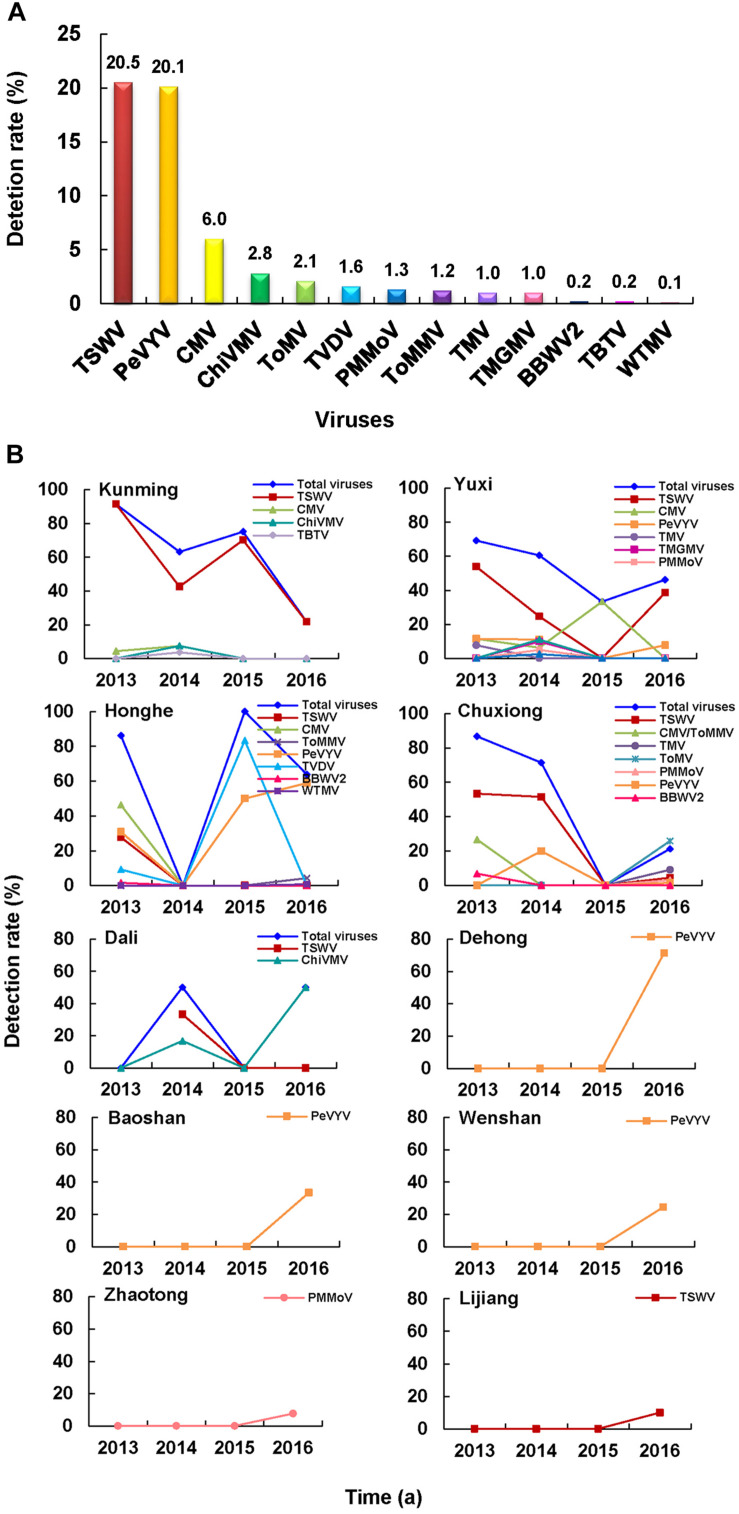
Pepper virome in Yunnan Province. **(A)** Detection rates of viruses in the pepper samples. **(B)** Distribution and dynamics of pepper-infecting viruses from 2013 to 2016.

A total of 13 RNA viruses were detected in 429 of the 821 assayed pepper samples, namely: TSWV, PeVYV, CMV, ChiVMV, ToMV, *Tobacco vein distorting virus* (TVDV), PMMoV, ToMMV, TMV, *Tobacco mild green mosaic virus* (TMGMV), BBWV2, TBTV, and *Wild tomato mosaic virus* (WTMV) (Figure 4A). Among these RNA viruses, TSWV and PeVYV were the most predominant viruses and were found in 20.5 and 20.1% of the assayed pepper samples, respectively (Figure 4A). In this study, pepper was first time found as a natural host of TVDV (GenBank accession no. MN889854). In addition, PeVYV (GenBank accession no. MN889851), TMGMV (GenBank accession no. MN889852), and WTMV (GenBank accession no. MN889853) were first time found in the pepper fields in Yunnan Province. Among the 49 pepper-infecting CMV isolates, 48 were CMV I subgroup isolates and one was CMV II subgroup isolate.

Of the 429 infected pepper samples, 51 samples were co-infected with two or three different viruses (i.e., CMV + TSWV, CMV + PeVYV, CMV + ToMMV, CMV + TVDV, CMV + WTMV, TSWV + PeVYV, TSWV + BBWV2, TMV + ToMV, ToMV + PMMoV, TMGMV + PMMoV, PeVYV + TVDV, CMV + ToMMV + PMMoV, CMV + TSWV + PeVYV, CMV + TVDV + PeVYV, and TMV + ToMV + PeVYV) (Supplementary Table 2). Among these mixed infections, CMV + TSWV and TSWV + PeVYV were most common combinations. The mixed infection of CMV + TSWV occurred mainly in Chuxiong, Honghe, Yuxi, and Kunming regions, while the mixed infection of TSWV + PeVYV occurred only in Honghe and Yuxi regions. Also, among these 15 co-infected types, eight had CMV and four contained TSWV (Supplementary Table 2).

TSWV was the most common virus in Kunming, Yuxi, and Chuxiong regions, while PeVYV was the most dominant virus in Honghe, Dehong, Baoshan, and Wenshan regions (Figure 4B). In Dali region, the most common virus was ChiVMV, while only PMMoV or TSWV was found in the pepper fields in Zhaotong and Lijiang regions (Figure 4B). The average detection rates of viruses in pepper samples collected in 2013, 2014, 2015, and 2016 were 77.7, 56.7, 75.5, and 38.8%, respectively. In general, the detection rate of TSWV in these years showed a downward trend, while the trend of PeVYV was in increase, especially in 2016 (Figure 4B).

### Distributions of Tomato-Infection Viruses

**FIGURE 5:**
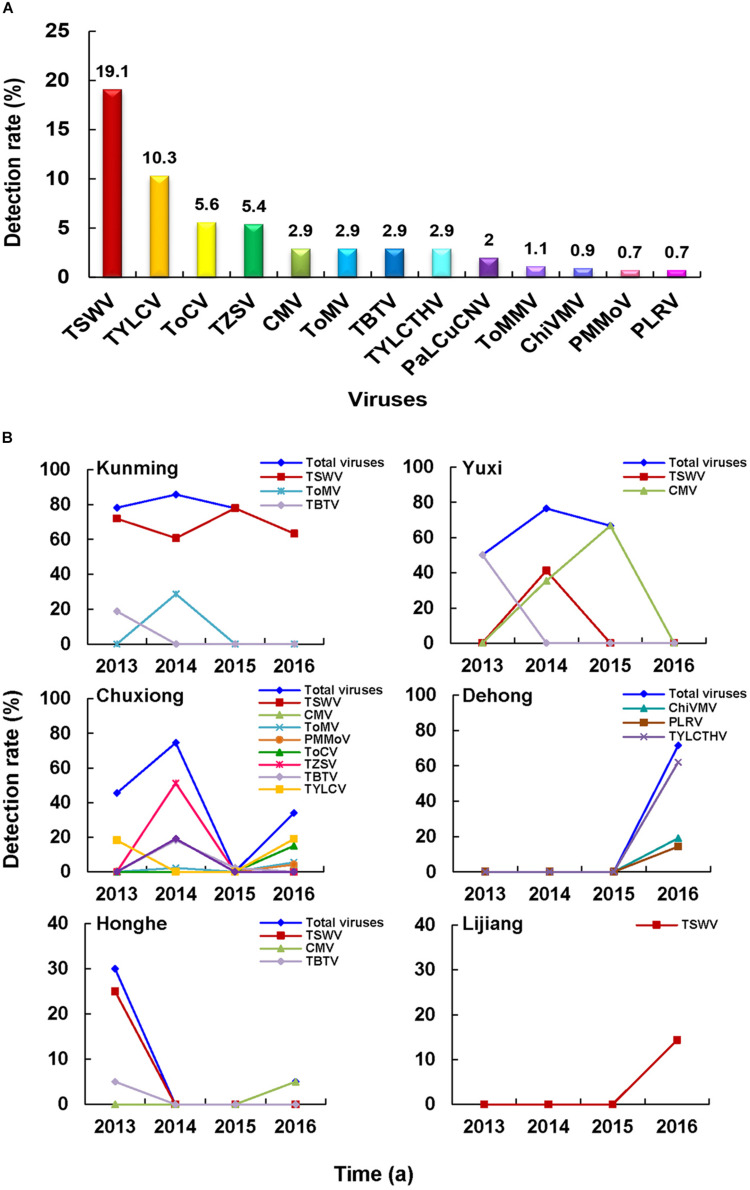
Tomato virome in Yunnan Province. **(A)** Detection rates of viruses in the tomato samples. **(B)** Distribution and dynamics of tomato-infecting viruses from 2013 to 2016.

A total of 10 RNA viruses and three DNA viruses were detected in 216 of the 446 assayed tomato samples (Figure 5A). The identified RNA viruses were TSWV, *Tomato chlorosis virus* (ToCV), *Tomato zonate spot orthotospovirus* (TZSV), CMV, ToMV, TBTV, ToMMV, ChiVMV, PMMoV, and *Potato leafroll virus* (PLRV), and the identified DNA viruses were *Tomato yellow leaf curl virus* (TYLCV), *Tomato yellow leaf curl Thailand virus* (TYLCTHV), and *Papaya leaf curl China virus* (PaLCuCNV). Among these viruses, TSWV and TYLCV were most common viruses found in the tomato samples. In this survey, PLRV (GenBank accession no. MN894826) was first time found in the infected tomato samples in China, while ToMMV (GenBank accession no. MN894824) and ChiVMV (GenBank accession no. MN894825) were first time found in the tomato samples in Yunnan Province. It is noteworthy that all the CMV isolates detected in the tomato samples belonged to the CMV II subgroup.

Among the 216 infected tomato samples, 38 were co-infected with two or three viruses (i.e., TSWV + ToMV, TSWV + TBTV, ToMV + PMMoV, TYLCV + ToCV, TYLCV + ToMMV, TYLCV + TBTV, TYLCTHV + ChiVMV, TYLCTHV + PLRV, TYLCV + ToCV + ToMMV, and TYLCTHV + ChiVMV + PLRV) (Supplementary Table 3). Among these mix-infected samples, the most samples contained begomoviruses. The TYLCV + ToCV mixed infection was mainly found in the tomato fields collected from Chuxiong region, followed by TSWV + TBTV and then TYLCV + ToMMV mixed infection (Supplementary Table 3).

The dominant tomato-infecting viruses in different regions varied. For instance, TSWV was the most common tomato-infecting virus in Kunming region from 2013 to 2016, whereas CMV and TYLCV were the most common viruses in Yuxi and Chuxiong regions, respectively (Figure 5B). In 2013 and 2014, virus diseases were more common in Kunming, Yuxi, and Chuxiong regions, Honghe region in 2013, and Dehong region in 2016 (Figure 5B).

### Correlation Between Disease Symptoms and the Causal Viruses

In this study, the most common disease symptoms observed on the infected pepper and tomato plants were leaf mottling or mosaic, distortion, necrosis and yellowing, and plant stunting. To investigate the potential correlations between disease symptoms and the 19 identified viruses, we compared disease symptom and the virus identification data obtained in this study. The result indicated that the correlations were complex. Same virus often caused different disease symptoms in these two host plants of different varieties at different growth stages and in different infection seasons. In contrast, some different viruses caused similar disease symptoms in different infected pepper or tomato plants of the same variety. Furthermore, disease symptoms caused by different combinations of viruses in the pepper and tomato plants also varied and were influenced by the environmental conditions. Therefore, disease symptoms shown on infected plants cannot be directly linked to the causal virus(es). However, disease symptoms caused by a single certain virus in a particular host plant were similar, including blistering and ring spots in the TSWV-infected leaves or leaf distortion (i.e., shoestring) in CMV-infected leaves. Disease symptoms caused by viruses belonging to the same genus (i.e., TMV, ToMV, ToMMV, PMMoV, and TMGMV) were similar (i.e., mottling and mosaic in leaves).

## Discussion

In this study, we surveyed pepper and tomato fields in different regions in Yunnan Province, China (Figure 1A). The result showed that the disease severities in different regions varied significantly. A total of 19 viruses were identified in assayed pepper and tomato samples collected in this study (Figures 2A,B). This number was higher than that previously reported for other regions in China (Wen et al., 2009; Wang et al., 2014, 2017b; Guo et al., 2015; Liu, 2015; Wang L. S. et al., 2015; Gao et al., 2016; Zhang et al., 2017b) and indicated that the virus population structures in the pepper and tomato fields in this region were more complex than that in the other regions of China. Our result showed that TSWV, PeVYV, CMV, and PMMoV occurred every year in the pepper and tomato fields in this province from 2013 to 2016 (Figure 2C). Among these four viruses, the occurrence of TSWV and CMV showed a decline trend, while the occurrence of PeVYV was gradually increasing. The occurrence of PMMoV maintained relatively stable from 2013 to 2016. Other viruses occurred sporadically in this period. During this survey, 13 viruses were detected in 2016 (Figure 2C), which can be useful for the design and development of pepper and tomato virus disease management strategy in this region.

The occurrence of TSWV in the pepper and tomato fields in 2013–2016 showed a downward trend (Figures 2C, 4B, 5B), probably due to the new control strategy designed for this virus in this area since 2014 (Zheng et al., 2015), which indicated that timely prevention and control was effective for virus disease management. The virus distributions in the pepper and tomato fields observed in the study (Figures 4B, 5B) revealed that virus diseases were more common in Chuxiong, Yuxi, Kunming, and Honghe regions than other regions, and the virus population structures in different surveyed regions varied (Figures 4B, 5B). For example, TSWV was the most common virus in Kunming and Yuxi regions, while PeVYV was a more common virus in Honghe, Baoshan, and Wenshan regions. Because different viruses are transmitted *via* different ways, management strategies for different regions should also vary accordingly. For example, applications of insect pesticides and resistant pepper and tomato varieties should be used in Kunming, Dehong, Honghe, Dali, and Baoshan regions because most dominant viruses in these regions are transmitted by insect vectors (Figure 3B). In contrast, a more integrated management measure should be used in Chuxiong region because this region has multiple viruses but none of them is prominent (Figure 3B).

Several recent studies have shown that CMV, tobamoviruses (i.e., TMV, ToMV, and/or PMMoV), and TYLCV were the major viruses in pepper and tomato fields in different provinces of China (Wen et al., 2009; Wu et al., 2013; Wang et al., 2014, 2017b; Guo et al., 2015; Liu, 2015; Wang L. S. et al., 2015; Gao et al., 2016; Liu et al., 2019). The results presented in this study showed that the virus population structures in the pepper and tomato fields in Yunnan Province changed in these years compared with an earlier report (Zhang et al., 1998). In that report, Zhang et al. (1998) had indicated that the main viruses in the pepper and tomato fields in this province were CMV and TMV. Due to the limitation of detection technology in that time, we cannot rule out the possibility of existence of other viruses in that study. TSWV is currently the most important virus in both pepper and tomato fields in Yunnan Province (Figures 4A, 5A) and has severe pathogenicity (Figures 1B,C) and very wide host range (Li et al., 2015; Xiao et al., 2015). The infection rates of CMV, TMV, ToMV, PMMoV, and TYLCV in Yunnan Province have declined in recent years. This reduction may be caused by the fact that the corresponding control measures such as virus-resistant varieties were made in the management of these viruses based on the previous reports in the province (Zhang et al., 1998; Zhang,2010). This report indicated that regular survey of the virus diseases was very important in management of viral diseases. Currently, CMV II subgroup isolates are more common in Yunnan Province than in other areas of China. Although both CMV I and II subgroup isolates are present in Yunnan Province, CMV I subgroup isolates are more common in the pepper fields, while CMV II subgroup isolates are more common in the tomato fields. The dominance of CMV I subgroup isolates in the pepper fields is consistent with the report published previously (Li et al., 2008b). The dominance of CMV II subgroup isolates in tomato fields has not been reported previously in other regions of China. It was reported that CMV I subgroup isolates were common in other vegetable crops in China (Chen et al., 2016) and CMV II subgroup isolates were common in the tobacco, lily, and tomato in Yunnan Province (Li et al., 2000; Zhao et al., 2007).

TBTV and TVDV were known to infect tobacco to cause bushy top disease (Wang D. Y. et al., 2015), it may be a challenge for the prevention and control of tobacco bush top disease, since these two viruses are newly identified in pepper and/or tomato fields in Yunnan Province (Li et al., 2018a). PeVYV is a new virus in the pepper fields in this province and is gradually becoming a dominant virus (Figure 4A). Its infection rate in pepper fields in 2016 has reached 19.8% (Figure 2C). The first report of ToMMV in the pepper fields in Yunnan Province was in 2014 (Li et al., 2014) and is now in the pepper, tomato, and eggplant in other parts of China (Chai et al., 2018; Li et al., 2020). Because ToMMV can cause severe diseases in many crops and its infection rate in vegetable crops is gradually increasing in China (Sui et al., 2017; Nagai et al., 2019; Li et al., 2020), it may become a devastating virus in many vegetable crops in China. We consider that more investigations are needed to identify new viruses so that specific virus resistance can be integrated in the pepper and tomato breeding programs.

In this survey, no virus was detected in some samples with virus-like symptoms. It is possible that these virus-like symptoms were induced by other factors, including fungi, insects, abiotic stresses, environmental conditions, and/or pesticides. It is also possible that the causal virus(es) were not covered by the detection methods. As the sample sizes from some locations were small, the disease incidence in these locations needs further validation. Nevertheless, the information presented here should allow a better management of virus diseases in Yunnan Province.

## Conclusion

We have conducted a comprehensive survey of virus diseases in the pepper and tomato fields in Yunnan Province, one of the main vegetable production provinces in China. Our result indicates that the virus population structure in the pepper fields is more complex than that in the tomato fields. This information is useful for a better management of virus diseases in these two vegetable fields in this region.

## Data Availability Statement

The datasets presented in this study can be found in online repositories. The names of the repository/repositories and accession number(s) can be found in the article/Supplementary Material.

## Author Contributions

YLi, GT, and FL conceived and designed the experiments. YLi, LX, and WZ performed the experiments. YLi and GT collected the samples and analyzed the data. PL, XC, and YLiu contributed reagents, materials, and analysis tools. YLi, RL, and FL wrote the manuscript. All authors read and approved the final manuscript.

## Conflict of Interest

The authors declare that the research was conducted in the absence of any commercial or financial relationships that could be construed as a potential conflict of interest.

## Footnotes

1 https://blast.ncbi.nlm.nih.gov/Blast.cgi

## Supplementary Material

Supplementary Table 1 Primers used in this study to detect viruses in pepper and tomato samples.

Supplementary Table 2 Numbers and detection rates of different viruses in the mix-infected pepper samples collected from different regions in this study.

Supplementary Table 3 Numbers and detection rates of different viruses in the mix-infected tomato samples collected from different regions in this study.
