# Supplementary material for: A Multiyear Survey and Identification of Pepper- and Tomato-Infecting Viruses in Yunnan Province, China
Source: Front Microbiol. 2021 Feb 26;12:623875. doi: 10.3389/fmicb.2021.623875 (PMC7953161; doi:10.3389/fmicb.2021.623875)
Supplement: Supplementary Table 1 — Primers used in this study to detect viruses in pepper and tomato samples. [file Data_Sheet_1.docx]

**Supplementary Table 1** Primers used in this study to detect viruses in pepper and tomato samples

| **Target Virus^a^/Viruses** | **Primer Name** | **Sequence (5’ – 3’)** | **Size of Amplicon (bp)** | **Tm (ºC)** | **Reference** |
| --- | --- | --- | --- | --- | --- |
| begmoviruses | BegoAFor1 | TGYGARGGICCITGYAARGTYCARTC | 1200 | 40 | Ha et al. 2006 |
|  | BegoARev1 | ATHCCMDCHATCKTBCTITGCAATCC |  |  |  |
| potyviruses | CIFor | GGIVVIGTIGGIWSIGGIAARTCIAC | 700 | 40 | Ha et al. 2008 |
|  | CIRev | ACICCRTTYTCDATDATRTTIGTIGC |  |  |  |
| poleroviruses | PoconF | GAYTGYTCYGGTTTTGACTGG | 1394 | 57 | Zhou et al. 2011 |
|  | PocoCPR | CGTCTACCTATTTSGGRTTN |  |  |  |
| tobamoviruses | TobamodF | TKGAYGGNGTBCCNGGNTGYGG | 880 | 55 | Li et al. 2018c |
|  | TobamodR | ACNGAVTBNABCTGTAATTGCTAT |  |  |  |
| TMV | TMVF | CGGTCAGTGCCGAACAAGAA | 693 | 55 | This study |
|  | TMVR | ATTTAAGTGGASGGAAAAVCACT |  |  |  |
| ToMV | ToMVF | GCGGAAGGCCTAAACCAAAAAG | 704 | 57 | This study |
|  | ToMVR | CAAACTTTATATTTCAGCACCTATGC |  |  |  |
| PMMoV | PMMoVF | GTAAGAGAAATGATAATAAGGGTTTG | 728 | 55 | This study |
|  | PMMoVR | CGTTCGCAAATACACGTCAC |  |  |  |
| TMGMV | TMGMVdF | GAGGAAATTGAGGATAATGTAAGTG | 700 | 55 | This study |
|  | TMGMVdR | ACGCCATACCACAGTATACAC |  |  |  |
| ToMMV | ToMMVdF | CTGGAGAAGACTGGGTCTAG | 1193 | 55 | Li et al. 2020 |
|  | ToMMVdR | TTCGGTAAGTTCAATGGGACCT |  |  |  |
| ChiVMV | ChiVMVdF | GGATAGAGCTGARCARCCAG | 920 | 57 | Li et al. 2018b |
|  | ChiVMVdR | CTTTGAAGCCCATATCTTGGC |  |  |  |
| CMV | CMVCPuF | TCTCATGGATGCTTCTCCGCG | 760 | 55 | Li et al. 2018b |
|  | CMVCPuR | CCGTAAGCTGGATGGACAACC |  |  |  |
| CMV I | CMVIdF1 | CCGAAGTAACCCAYGGTCGT | 969 | 55 | This study |
|  | CMVIdR1 | GATTTGTCCATGACTCGACTC |  |  |  |
| CMV II | CMVIIdF2 | CGCGAGTTAGCGTTTAGTTGT | 762 | 55 | This study |
|  | CMVIIdR2 | TTAACGTCTTCGGACGCCG |  |  |  |
| TSWV | TSWVF | TCACTGTAATGTTCCATAGCAA | 861 | 52 | Liu et al. 2019 |
|  | TSWVR | AGAGCAATYGTGTCAATTTTATTC |  |  |  |
| TZSV | TZSVdF | TGGTTAAAAAGACAGATCATTGCT | 852 | 55 | This study |
|  | TZSVdR | CTACTTGCCAACATGTCTAACGTC |  |  |  |
| ToCV | ToCVCPF | GAATCTTTTAGAAGCTTTGGTTTAAGG | 848 | 57 | Hirota et al. 2010 |
|  | ToCVCPR | GATCCTCTTGATCCTCATAGATTTC |  |  |  |
| BBWV2 | BBWV2dF | AGAAAYAGGAAGGTGCGTGC | 1656 | 53 | Zhang et al. 2017b |
|  | BBWV2dR | TCTRTTGCACATGGCATACC |  |  |  |
| PeVYV | PeVYVdF | CGTGGAAGCGTGCTACTCG | 579 | 57 | Liu et al. 2019 |
|  | PeVYVdR | CTCATCAGTGAAGACTCGACC |  |  |  |
| PLRV | PLRVdF | CCAAGCATACGCGAGTTGC | 899 | 55 | Liu et al. 2019 |
|  | PLRVdR | GTATGCCAACGTAAGCAATAAATC |  |  |  |
| TBTV | TBTVdF | TACCACACCTAAACAGCGTTG | 1049 | 55 | Liu et al. 2019 |
|  | TBTVdR | CTCATCTCCCGCTAAGTCAG |  |  |  |
| TVDV | TVDVdF | GCAACAGCGAGACTTTCATCT | 357 | 56 | Liu et al. 2019 |
|  | TVDVdR | CRTTGCCTTTATAGAGCAGCC |  |  |  |
| AMV | AMVdF | GTGCGTATAGATGCCGGTTC | 900 | 55 | Liu et al. 2019 |
|  | AMVdR | GAGCGAATAGGACTTCATACC |  |  |  |
| MPV | MPVdF | GACCGTTCTCACCTTGACG | 924 | 55 | Liu et al. 2019 |
|  | MPVdR | CAGTGCAAGTAACCCACATGC |  |  |  |
| TAV | TAVdF | ATGGCCCAAAACGGTACGG | 657 | 57 | Liu et al. 2019 |
|  | TAVdR | TCACACCGGGAGCGTTGAAG |  |  |  |

^a^ TMV - *Tobacco* mosaic virus; ToMV - *Tomato mosaic virus*; PMMoV - *Pepper mild mottle virus*; TMGMV - *Tobacco mild green mosaic virus*; ToMMV - *Tomato mottle mosaic virus*; ChiVMV - *Chilli veinal mottle virus*; CMV - *Cucumber mosaic virus*; CMV I - *Cucumber mosaic virus* subgroup I; CMV II - *Cucumber mosaic virus* subgroup II; TSWV - *Tomato spotted wilt orthotospovirus*; TZSV - *Tomato zonate spot orthotospovirus*; ToCV - *Tomato chlorosis virus*; BBWV2 - *Broad bean wilt virus 2*; PeVYV - *Pepper vein yellows virus*; PLRV - *Potato leafroll virus*; TBTV - *Tobacco bushy top virus*; TVDV - *Tobacco vein distorting virus*; AMV - *Alfalfa mosaic virus*; MPV - *Moroccan pepper virus*; TAV - *Tomato aspermy virus*.

**Supplementary Table 2** Numbers and detection rates of different viruses in the mix-infected pepper samples collected from different regions in this study

| Sampling areas | Total samples | CMV + TSWV | CMV+  PeVYV | CMV+  ToMMV | CMV+  TVDV | CMV+  WTMV | TSWV+  PeVYV | TSWV+  BBWV2 | TMV+  ToMV | ToMV+  PMMoV | TMGMV+  PMMoV | PeVYV+  TVDV | CMV+  ToMMV+  PMMoV | CMV+  TSWV+  PeVYV | CMV+  TVDV+  PeVYV | TMV+  ToMV+  PeVYV | Number of  positive samples | Detection rate (%)^a^ |
| --- | --- | --- | --- | --- | --- | --- | --- | --- | --- | --- | --- | --- | --- | --- | --- | --- | --- | --- |
| Chuxiong | 116 | 6 | 0 | 3 | 0 | 0 | 0 | 0 | 5 | 2 | 0 | 0 | 1 | 0 | 0 | 1 | 18 | 15.52 |
| Honghe | 212 | 4 | 5 | 0 | 0 | 1 | 9 | 1 | 0 | 0 | 0 | 2 | 0 | 0 | 0 | 0 | 22 | 10.38 |
| Yuxi | 135 | 2 | 0 | 0 | 1 | 0 | 4 | 0 | 0 | 0 | 1 | 0 | 0 | 1 | 1 | 0 | 10 | 7.41 |
| Kunming | 140 | 1 | 0 | 0 | 0 | 0 | 0 | 0 | 0 | 0 | 0 | 0 | 0 | 0 | 0 | 0 | 1 | 0.71 |
| Dali | 28 | 0 | 0 | 0 | 0 | 0 | 0 | 0 | 0 | 0 | 0 | 0 | 0 | 0 | 0 | 0 | 0 | 0 |
| Dehong | 11 | 0 | 0 | 0 | 0 | 0 | 0 | 0 | 0 | 0 | 0 | 0 | 0 | 0 | 0 | 0 | 0 | 0 |
| Wenshan | 89 | 0 | 0 | 0 | 0 | 0 | 0 | 0 | 0 | 0 | 0 | 0 | 0 | 0 | 0 | 0 | 0 | 0 |
| Baoshan | 51 | 0 | 0 | 0 | 0 | 0 | 0 | 0 | 0 | 0 | 0 | 0 | 0 | 0 | 0 | 0 | 0 | 0 |
| Lijiang | 10 | 0 | 0 | 0 | 0 | 0 | 0 | 0 | 0 | 0 | 0 | 0 | 0 | 0 | 0 | 0 | 0 | 0 |
| Zhaotong | 13 | 0 | 0 | 0 | 0 | 0 | 0 | 0 | 0 | 0 | 0 | 0 | 0 | 0 | 0 | 0 | 0 | 0 |
| Qujing | 13 | 0 | 0 | 0 | 0 | 0 | 0 | 0 | 0 | 0 | 0 | 0 | 0 | 0 | 0 | 0 | 0 | 0 |
| Puer | 3 | 0 | 0 | 0 | 0 | 0 | 0 | 0 | 0 | 0 | 0 | 0 | 0 | 0 | 0 | 0 | 0 | 0 |
| Total number | 821 | 13 | 5 | 3 | 1 | 1 | 13 | 1 | 5 | 2 | 1 | 2 | 1 | 1 | 1 | 1 | 51 | 6.21 |
| Detection rate (%)^b^ |  | 1.58 | 0.61 | 0.37 | 0.12 | 0.12 | 1.58 | 0.12 | 0.61 | 0.24 | 0.12 | 0.24 | 0.12 | 0.12 | 0.12 | 0.12 |  |  |

^a^ Detection rate (%): number of positive samples / number of total samples in a certain sampling area.

^b^ Detection rate (%): number of samples infecting a certain virus / number of total samples of all sampling areas (821).

**Supplementary Table 3** Numbers and detection rates of different viruses in the mix-infected tomato samples collected from different regions in this study

| Sampling areas | Sample number | TSWV+  ToMV | TSWV+  TBTV | ToMV+  PMMoV | TYLCV+ ToCV | TYLCV +ToMMV | TYLCV+ TBTV | TYLCTHV+ ChiVMV | TYLCTHV+ PLRV | TYLCV+ ToCV+ToMMV | TYLCTHV+ ChiVMV+PLRV | Number of positive samples | Detection rate (%)^a^ |
| --- | --- | --- | --- | --- | --- | --- | --- | --- | --- | --- | --- | --- | --- |
| Dehong | 21 | 0 | 0 | 0 | 0 | 0 | 0 | 2 | 1 | 0 | 1 | 4 | 19.05 |
| Chuxiong | 190 | 0 | 1 | 3 | 19 | 4 | 1 | 0 | 0 | 1 | 0 | 29 | 15.26 |
| Kunming | 97 | 1 | 4 | 0 | 0 | 0 | 0 | 0 | 0 | 0 | 0 | 5 | 5.15 |
| Yuxi | 22 | 0 | 0 | 0 | 0 | 0 | 0 | 0 | 0 | 0 | 0 | 0 | 0 |
| Lijiang | 22 | 0 | 0 | 0 | 0 | 0 | 0 | 0 | 0 | 0 | 0 | 0 | 0 |
| Honghe | 60 | 0 | 0 | 0 | 0 | 0 | 0 | 0 | 0 | 0 | 0 | 0 | 0 |
| Wenshan | 25 | 0 | 0 | 0 | 0 | 0 | 0 | 0 | 0 | 0 | 0 | 0 | 0 |
| Baoshan | 6 | 0 | 0 | 0 | 0 | 0 | 0 | 0 | 0 | 0 | 0 | 0 | 0 |
| Qujing | 3 | 0 | 0 | 0 | 0 | 0 | 0 | 0 | 0 | 0 | 0 | 0 | 0 |
| Total number | 446 | 1 | 5 | 3 | 19 | 4 | 1 | 2 | 1 | 1 | 1 | 38 | 8.52 |
| Detection rate (%) |  | 0.22 | 1.12 | 0.67 | 4.26 | 0.90 | 0.22 | 0.45 | 0.22 | 0.22 | 0.22 |  |  |

^a^ Detection rate (%) : number of positive samples / number of total samples in a certain sampling area.

^b^ Detection rate (%) : number of samples infecting a certain virus / number of total samples of all sampling areas (446).
